# Interannual Changes in Biomass Affect the Spatial Aggregations of Anchovy and Sardine as Evidenced by Geostatistical and Spatial Indicators

**DOI:** 10.1371/journal.pone.0135808

**Published:** 2015-08-27

**Authors:** Marco Barra, Pierre Petitgas, Angelo Bonanno, Stylianos Somarakis, Mathieu Woillez, Athanasios Machias, Salvatore Mazzola, Gualtiero Basilone, Marianna Giannoulaki

**Affiliations:** 1 Institute for Coastal and Marine Environment, Detached Units of Capo Granitola, Mazara del Vallo and Naples, Italy; 2 IFREMER, Laboratoire d’Ecologie Halieutique, Nantes, France; 3 Hellenic Centre for Marine Research, Institute of Marine Biological Resources and Inland Waters, Iraklion, Greece; Aristotle University of Thessaloniki, GREECE

## Abstract

Geostatistical techniques were applied and a series of spatial indicators were calculated (occupation, aggregation, location, dispersion, spatial autocorrelation and overlap) to characterize the spatial distributions of European anchovy and sardine during summer. Two ecosystems were compared for this purpose, both located in the Mediterranean Sea: the Strait of Sicily (upwelling area) and the North Aegean Sea (continental shelf area, influenced by freshwater). Although the biomass of anchovy and sardine presented high interannual variability in both areas, the location of the centres of gravity and the main spatial patches of their populations were very similar between years. The size of the patches representing the dominant part of the abundance (80%) was mostly ecosystem- and species-specific. Occupation (area of presence) appears to be shaped by the extent of suitable habitats in each ecosystem whereas aggregation patterns (how the populations are distributed within the area of presence) were species-specific and related to levels of population biomass. In the upwelling area, both species showed consistently higher occupation values compared to the continental shelf area. Certain characteristics of the spatial distribution of sardine (e.g. spreading area, overlapping with anchovy) differed substantially between the two ecosystems. Principal component analysis of geostatistical and spatial indicators revealed that biomass was significantly related to a suite of, rather than single, spatial indicators. At the spatial scale of our study, strong correlations emerged between biomass and the first principal component axis with highly positive loadings for occupation, aggregation and patchiness, independently of species and ecosystem. Overlapping between anchovy and sardine increased with the increase of sardine biomass but decreased with the increase of anchovy. This contrasting pattern was attributed to the location of the respective major patches combined with the specific occupation patterns of the two species. The potential use of spatial indices as auxiliary stock monitoring indicators is discussed.

## Introduction

Living organisms tend to aggregate into patches and thus seldom distribute randomly or uniformly in space [1]. In particular, small pelagic fish present aggregative behaviour at various scales, ranging from the individuals within schools to the spatial pattern of school patches or school clusters (e.g. [2], [3], [4]). A patch of schools could be pictured as concentric circles or ellipses of progressively increasing density from the periphery to the core. The spatial patterns of school clusters present special interest as they are related to fish catchability and fishing success [5]. As small pelagics hold an intermediate position in the marine food web, they can affect the aggregation patterns of both their prey and predators. Aggregation patterns may be controlled by environmental conditions, the distribution of food resources, the presence of conspecifics and competing species as well as density dependent effects [6], [7], [8]. Understanding those mechanisms that determine fish spatial distribution is not trivial and can often be quite complex.

Recently, a suite of indices have been developed to describe the spatial patterns observed at different scales, e.g. schools, clusters of schools or the spatial distribution at a population level [9]. School cluster indices involve the number of clusters, number of solitary schools, dimension of clusters, number of schools per unit cluster length, and the nearest neighbour distance within clusters [2]. More recently, a series of spatial indices have been proposed to identify how fish aggregations are geographically organized at population level, addressing characteristics such as the occupation, aggregation, dispersion, location, correlation and overlap [3].

Most geostatistical analyses of small pelagic fishes either involve geostatistical analysis in a single ecosystem (e.g. Mediterranean Sea: [9], [10], [11]); southern Africa: [12], Chile: [13]; Humboldt Current: [14], [15]; Bay of Biscay: [16], [17]; North Atlantic [18]; North Sea: [19]) or focus on the optimization of survey design in terms of spatial patterns (e.g. [11], [15]). Other studies limit their approach to aggregation curves (e.g.[16], [17]) and the study of the abundance-occupancy dynamics (e.g. [20], [21], [22]) rather than associating stock dynamics with specific spatial indices. Changes in the spatial structure have been reported for Northern cod, associating the autocorrelation range with the collapse of the stock [23]. In the southern Africa anchovy and sardine, only small changes in the indicator variograms have been found between years of high and low stock abundance [12]. The average size of school patches has also been shown to vary depending on the size and the topographic characteristics of the distribution area [10]. Recently, the use of indicator variograms has been proposed for estimating an acoustic index of abundance for two sardinella species [24]. Hence, assessing the relationship between spatial indices and stock abundance at different spatial scales and habitats can help to identify auxiliary stock status indices based on fish aggregation patterns.

This work aims making progress in this direction by studying the aggregation patterns of European anchovy (*Engraulis encrasicolus*) and sardine (*Sardina pilchardus*) in two selected ecosystems: the Strait of Sicily and the North Aegean Sea, both located in the Mediterranean basin. In the study areas, small pelagics are mainly fished using purse seines. The catchability of this fishing gear largely depends on the skills of the fishermen and good knowledge of the spatial patterns of fish in order to detect large aggregations of the target fish successfully [5]. Recent stock assessment reports show that during the last decade anchovy and sardine have suffered from high exploitation rates in the study areas and practically throughout the Mediterranean Sea [25], [26]. Acoustic surveys, conducted during summer since the early 00s, indicate that the respective anchovy and sardine populations differ between the two ecosystems both in terms of abundance and magnitude of their interannual variability [27]. Thus, identification of a possible relationship between the spatial patterns of species and biomass levels is of particular interest.

**Fig 1:**
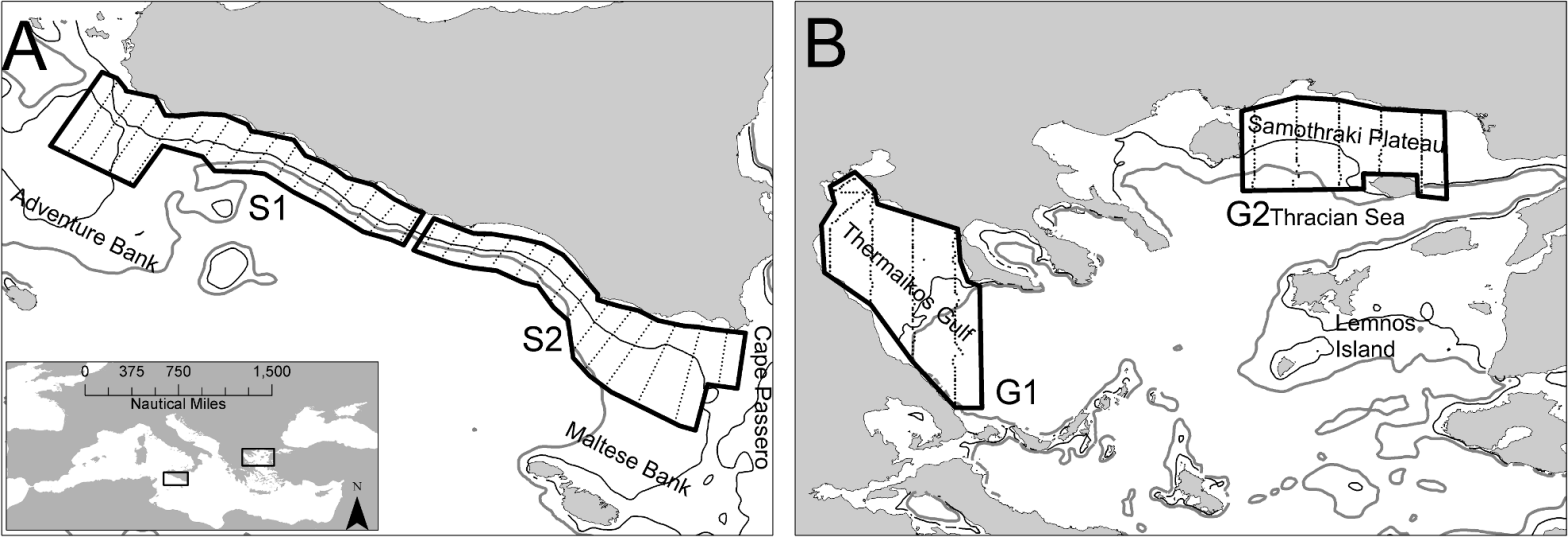
Study areas and sampling design in the Strait of Sicily (A, C) and in the North Aegean Sea (B, D). Sub-areas are also shown. S1: Adventure Bank, S2: Maltese Bank, G1: Thermaikos Gulf, G2: Thracian Sea

The two selected ecosystems largely differ in terms of environmental and topographic characteristics. The Strait of Sicily (Fig 1A) is an upwelling area with complex circulation patterns mainly determined by the motion of the Atlantic Ionian Stream. The latter produces a cyclonic vortex over Adventure Bank causing the formation of a permanent upwelling along the southern coast of Sicily. Wind-induced upwelling events often occurring in the coastal area reinforces the permanent upwelling process [28]. The intensity of the upwelling processes causes high interannual variability in primary production that also affects the interannual variability of small pelagic fish [27]. Anchovy and sardine biomass in the Strait of Sicily ranges from 3000 to 20000 t and from 6000 to 20000 t, respectively [27]. In contrast to the Strait of Sicily, the North Aegean Sea (Fig 1B) is a continental shelf area influenced by freshwater inflow. It is characterized by high hydrological complexity mostly related to low salinity Black Sea water that generates a permanent anticyclonic system in the eastern part of the area [29]. The latter, together with a number of rivers along the North Aegean Sea coastline, enhance local productivity [29] and subsequently affect the abundance of anchovy and sardine populations. In this area, anchovy and sardine biomass ranges from 25000 to 60000 t and 15000 to 40000 t, respectively [27].

In this study we used population spatial indices [3] to identify: i) the way in which fish aggregations are organized geographically in the two ecosystems; ii) any existing spatial patterns; iii) common traits in the spatial structures of the two species and iv) examine whether these traits are related to fish biomass.

## Material and Methods

### Acoustic data collection

Ethical approval and field permits were not required for this study because no animal samples were taken. We only used positional and acoustic data obtained from existing data sets.

All data used in this study come from echosurveys complying with the MEDIAS (Mediterranean International Acoustic Survey) protocol. Acoustic sampling was performed during day time by means of scientific split-beam echosounders working at 38 kHz and calibrated following standard techniques [30]. Acoustic data were recorded in both areas following a regular sampling design (parallel transects, Fig 1) at a constant speed of 8–10 nmi h^-1^. In the Strait of Sicily, acoustic data were collected during June–July 2002–2010 on board RV “G. Dallaporta”. The echosurvey sampling strategy adopted parallel transects characterized by 5 nmi inter-transect distance (Fig 1A). The investigated bathymetric range was 20–300 m. Acoustic data were acquired using Simrad EK500 (in 2002 and 2003) and Simrad EK60 (in 2005–2010) scientific split-beam echosounders. In this paper the analysis was limited to specific sub-areas, surveyed by applying a consistent survey design protocol (Fig 1A). In the North Aegean Sea, acoustic data were collected on board R/V Philia during June 2004–2006 and 2008 in two sub-areas: Thracian Sea and Thermaikos gulf (Fig 1B). Echosurveys were carried out along predetermined parallel transects with 10 nmi inter-transect distance, by means of a Biosonic DT-X scientific split-beam echosounder. Minimum sampling depth varied from 10 to 20 m depending on the area. The size of the Elementary Distance Sampling Unit (EDSU) was one nautical mile (nmi, 1.852 km). Midwater pelagic trawl sampling was carried out in order to identify and verify anchovy and sardine echo traces and fish length frequencies. We considered as anchovy/sardine any school or echo assigned either by echo trace classification or based on the catch composition of identification hauls [31]. Acoustic data analysis was made using Myriax Echoview software in all cases. Anchovy and sardine density (tnmi^-2^) for each EDSU was evaluated by merging the biological and acoustic data, based on the nearest haul method [31]. Daytime nautical area backscattering coefficients (NASC) assigned to anchovy/sardine were estimated for each ESDU. Further details on acoustic sampling per survey area are given below.

Total species biomass in the Strait of Sicily was computed for each year by assigning an area of influence to each EDSU, multiplying the density values of each EDSU by the corresponding area of influence and summing up all resulting values. In the North Aegean Sea, total species biomass was computed separately for each sub-area and year based on the abundance estimate on a rectangular grid as described in [31] by assigning an area of influence to each parallel transect, multiplying the mean density values of each transect with the corresponding area of influence and summing up all resulting values.

### Spatial analysis

**Fig 2:**
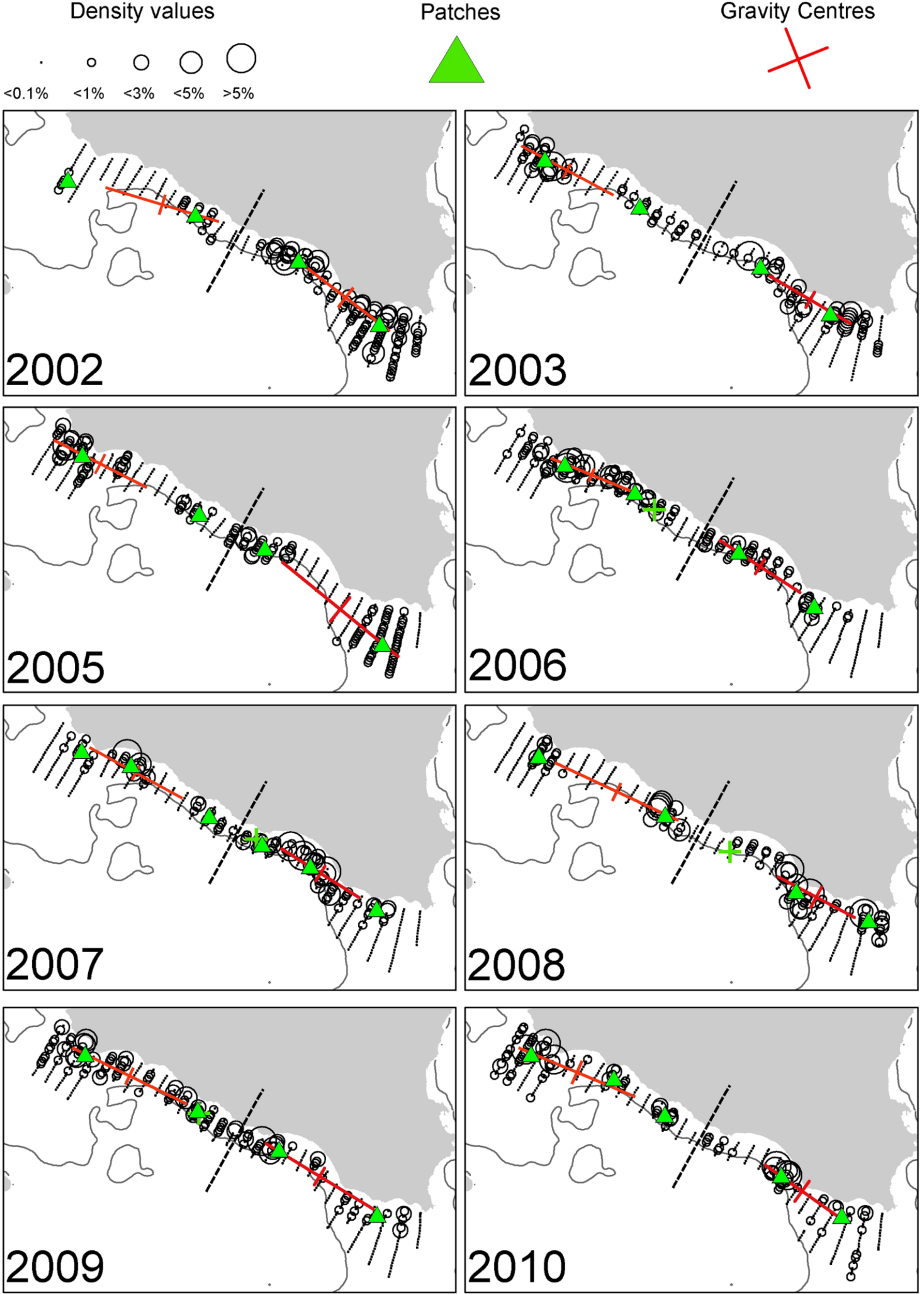
Annual maps of anchovy density distribution in the Strait of Sicily during summertime. The position of the centres of gravity and the main spatial patches (contributing > 10% to abundance) are shown. The 200 m isobath is indicated by a continuous line.

In order to describe the prominent spatial characteristics (position and space occupation) of anchovy and sardine populations, and highlight possible density-dependent effects, different spatial indicators were adopted taking into account different aspects of space occupation dynamics. Specifically, we applied geostatistical methodology (indicator variography analysis) to assess the geometric aspects of the fish patches contributing most to fish abundance and, in addition, we estimated a series of spatial indicators developed by [3] to quantify features such as occupation, aggregation, dispersion, location, and overlap at a larger spatial scale. Furthermore, the mean packing density (Pck, the ratio between total biomass and positive area) was calculated. All indicators were estimated per species and area. Based on preliminary spatial analysis, the study area of the Strait of Sicily was divided into two distinct sectors (S1 and S2 corresponding to Adventure and Maltese Bank respectively, separated by the dashed line shown in Fig 2).

#### Geostatistical analysis–Indicator variography

In order to capture and model the geometry of the spatial structures associated to high density fish patches (those mainly targeted by the fishery), we used the omnidirectional indicator variogram [32], [16]. Each density value was transformed into a binary variable representing classes of values, on the basis of a predetermined threshold. For this purpose, the cut-off value at 80% of total biomass (c80) was computed for each year and sub-area on the basis of the respective cumulative curves [17]. Denoting z(x) as the fish density at point x, Iz(x)>c80 equals 1 when the value z(x) is greater than the cut-off c80 and 0 otherwise. This c80 involves density values that allow recovering 80% of total biomass thus describing the spatial structure of the dominant part of fish abundance.

EVA2 software [33] was used to estimate the autocorrelation range (structural component) and the normalized nugget (i.e. expressed in percentage of variance) of the indicator variogram for each year, sub-area and target species. These variables represent an approximate measure of the average size of fish patches and small-scale roughness, respectively.

#### Spatial indicators

A series of spatial indicators was calculated on density values (tnmi^-2^) for anchovy and sardine in each study sub-area (Table 1). An exhaustive description of the derivation of each indicator and the functions used can be found in [3]. Spatial data are given in the WGS84 coordinate system. All indices were calculated by means of a set of R functions (available in electronic format at www.alr-journal.org (see [3], appendix S1). Analysis was performed using R v. 2.15.3 [34]. Although in each study area a regular sampling design was adopted, in some small sectors the design was not perfectly regular (e.g. the northern part of Thermaikos Gulf and the offshore sector of Maltese Bank) due mainly to the morphology of the coastline and the bathymetric gradient. Thus, all spatial indices were computed by weighting each EDSU with its area of influence in order to be able to include small sectors where the sampling design was not regular.

**Table 1:** Summary of the spatial indices (based on [3]) used to characterize the spatial pattern of anchovy and sardine populations.

Indicator	Short name	Units/Range	Associated population feature	Description
Centre of Gravity	CG	Degree	Location	Mean location of the population
Number of Patches	NP	>0	Patchiness	Patchiness, biomass hotspots
Inertia	I	nmi^2^	Dispersion	Dispersion of the individuals around the GC of the population
Isotropy	I	0 to 1	Dispersion	Elongation of the population
Positive Area	PA	nmi^2^	Occupation	Area of presence
Spreading Area	SA	nmi^2^	Aggregation	Area occupied by the population accounting for density values
Equivalent Area	EA	nmi^2^	Aggregation	Individual-based measure of the area occupied by the stock
Global Index of Collocation	GIC	0 to 1	Overlap	Overlapping degree of two populations

As mentioned previously, this suite of indicators were selected on the basis that they describe different aspects of medium-scale structures of anchovy and sardine. Specifically, the Centre of Gravity (CG) represents a positional index related to the mean location of the entire population [35]. The number of major spatial patches, or the number of biomass hotspots, was estimated using a threshold distance of 20 nmi (i.e. minimum distance from sample to patch centre) in both areas. Only spatial patches with an abundance of >10% of overall abundance were retained. Inertia and isotropy describe the dispersion of the population around its centre of gravity (i.e. the mean square distance between a random individual and the centre of gravity of the population) and whether such dispersion is characterized by isotropic behaviour (i.e. homogenous dispersion of the population in every direction), respectively. The Positive Area (PA) corresponds to the area occupied by the stock whereas Spreading Area (SA) and Equivalent Area (EA) measure specific aspects of aggregation and are related to each other [3],[35]. Finally, the Global Index of Collocation (GIC, [3]) is a measure of the spatial overlap between anchovy and sardine populations. The index ranges from 0 when each population is concentrated in a single but different location to 1, when the two *CG*s coincide [3].

#### Principal Component Analysis

In a next step of the analysis, we examined the relationship between biomass and a suite of spatial indicators instead of single indicators. All indicators were standardized (i.e. each value was divided by the species and area mean) except for the number of major patches and the CG coordinates (CGy, CGx). Subsequently, Principal Component Analysis (PCA) [36] was applied to the correlation matrix of all spatial indicators as well as the range and the percentage of the nugget from the indicator variograms (active variables). Specifically, PCA was applied to: a) pooled anchovy data from both areas; b) pooled sardine data from both areas; c) pooled species data from the Strait of Sicily and d) pooled species data from the North Aegean Sea. Analysis per both area and species was not possible due to the limited number of available data (years).

Standardized fish abundance (abundance divided by the mean in the area) and Pck were added as supplementary variables [36]. Such a procedure allows identification of possible correlations between supplementary and active variables. R library FactoMineR [37] software was used for the analysis, a package for multivariate data analysis with R [34], available from the Comprehensive R Archive Network (CRAN) at http://CRAN.R-project.org/.

#### Multiple Regression Analysis

In a subsequent step, multiple regression was applied to relate the standardized abundance of anchovy and sardine with the PCA scores of the most important PCA axes (i.e. explaining ~85 to 95% of the variance). A stepwise procedure was applied to select those PCA axes significantly entering into the regression models. The presence of outliers as well as the linearity of the relationships was assessed by inspecting the residual properties (randomly distributed, homoscedastic, lack of trend).

## Results

### Distribution and biomass

In the Strait of Sicily, anchovy was more patchily distributed than sardine being more abundant in the western and the eastern part of the study area (over the Adventure and Maltese Banks, Fig 2). Sardine was mainly located in the narrow band of the central sector of the Strait of Sicily as well as over the Maltese Bank (occupying a large part of the western part only in 2009, Fig 3). Anchovy showed the lowest densities over Adventure Bank in 2002 and 2008. In these years, high anchovy densities were recorded in the coastal sector of the Maltese Bank mainly. In the North Aegean Sea, both species showed consistently higher density values in the northern part of Thermaikos Gulf. Higher variability in species distribution patterns was observed in the Thracian Sea where sardine exhibited very dense aggregations and patchier distribution compared to anchovy (Figs 4 and 5).

**Fig 3:**
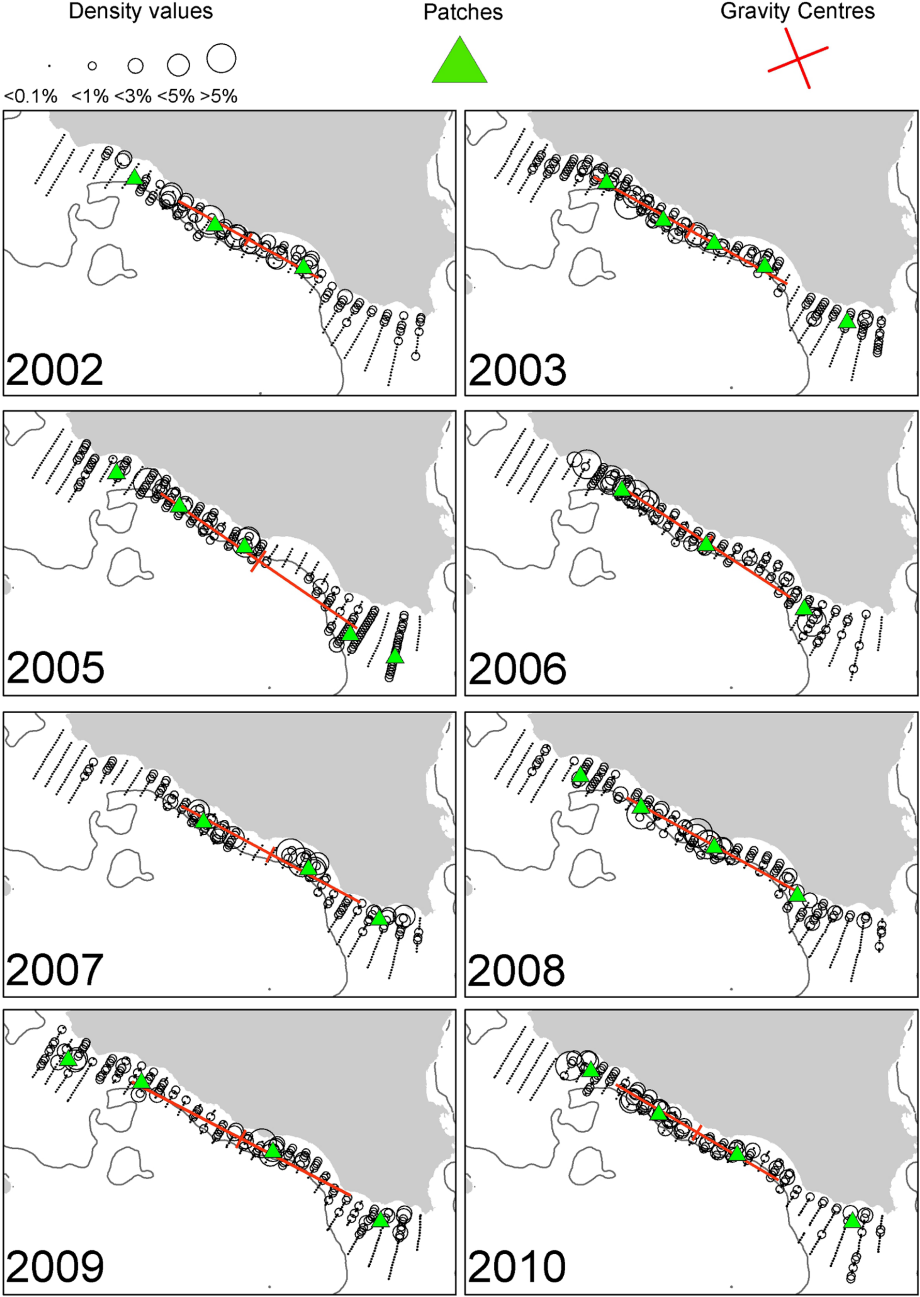
Annual maps of sardine density distribution in the Strait of Sicily during summertime. The position of the centres of gravity and the main spatial patches (contributing >10% to total abundance) are shown. The 200 m isobath is indicated by a continuous line.

**Fig 4:**
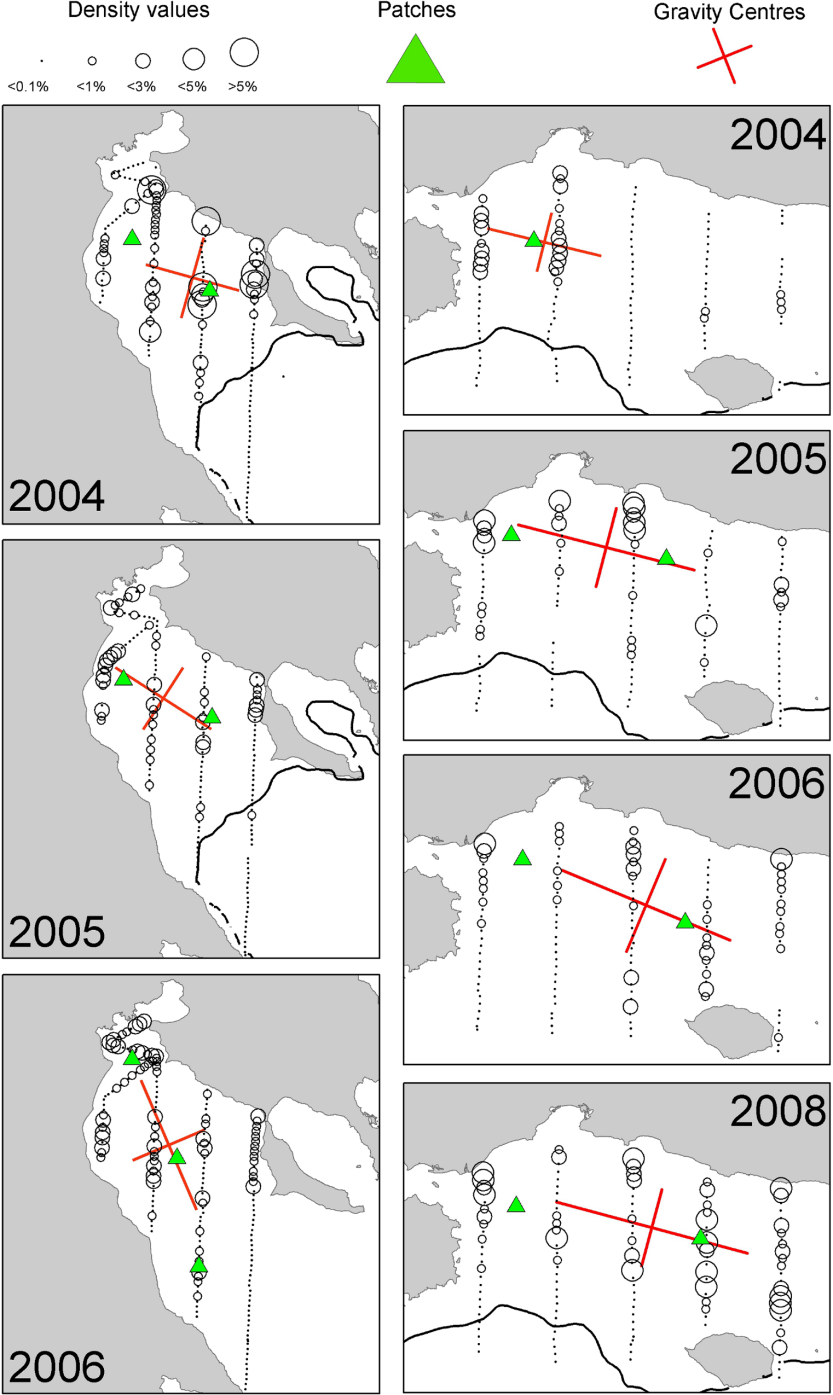
Annual maps of anchovy density distribution in the North Aegean Sea during summertime. The position of the centres of gravity and the main spatial patches (contributing >10% to total abundance) are shown. The 200 m isobath is indicated by a continuous line.

**Fig 5:**
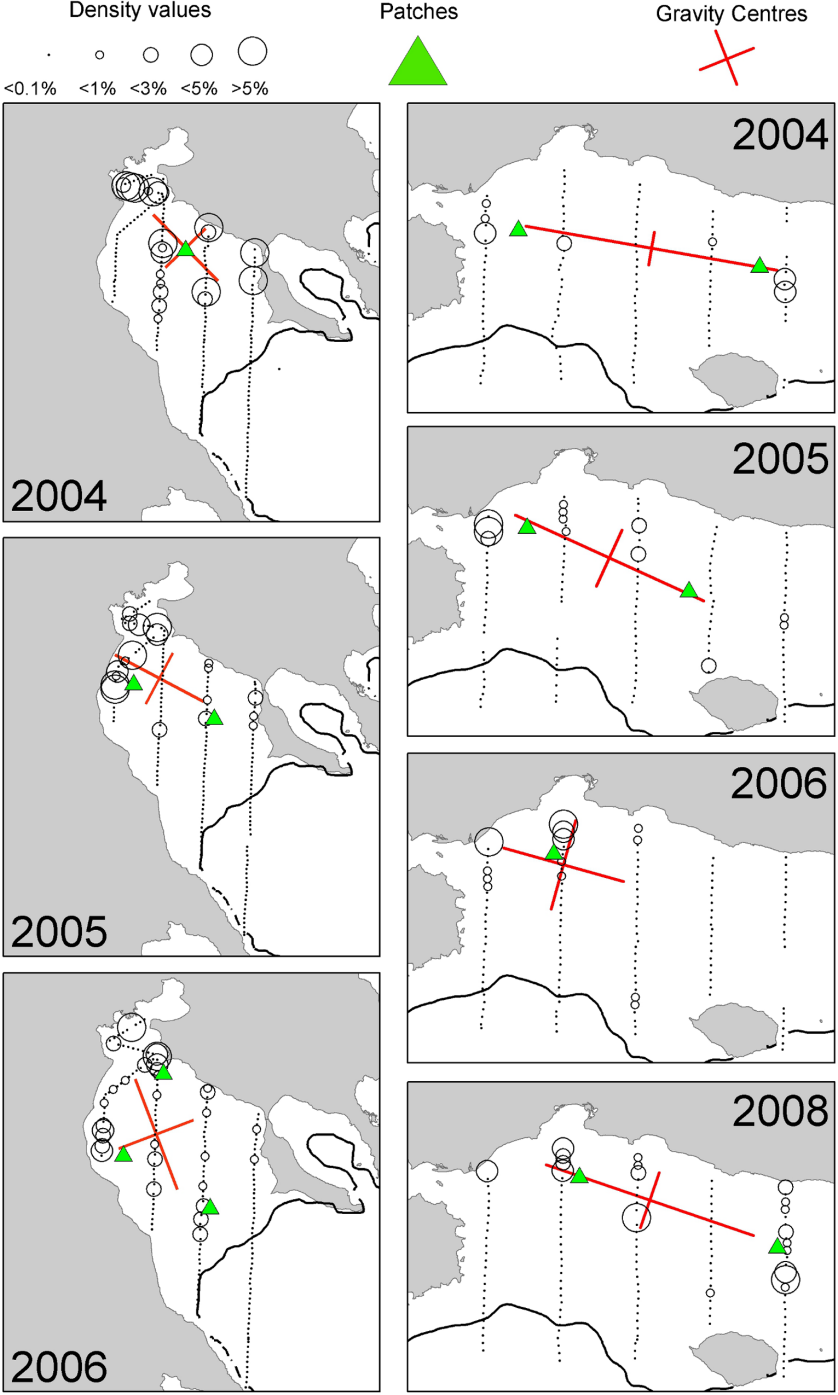
Annual maps of sardine density distribution in the North Aegean Sea during summertime. The position of the centres of gravity and the main spatial patches (contributing >10% to total abundance) are shown. The 200 m isobath is indicated by a continuous line.

**Fig 6:**
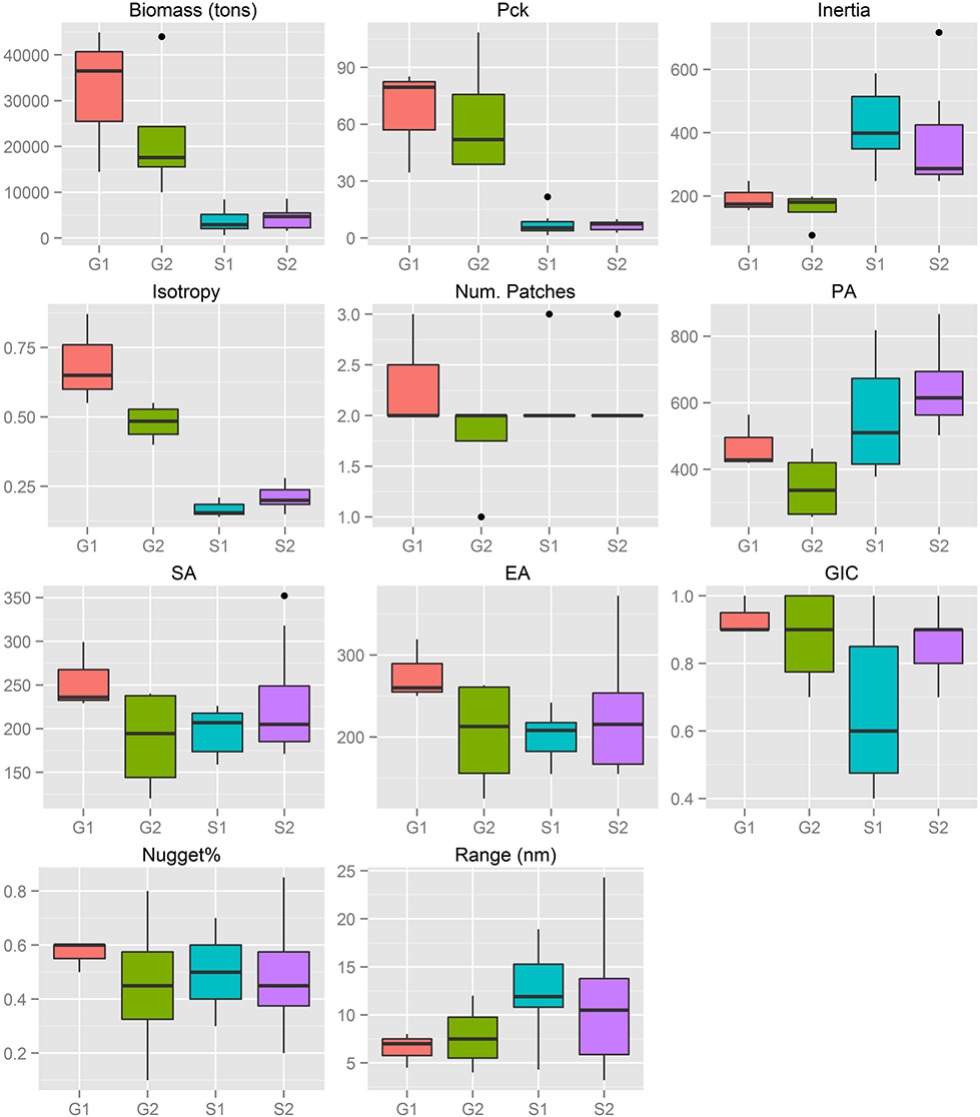
Anchovy: Box-plots of biomass (t), packing density (Pck) and spatial indicator values per sub-area. See Table 1 for descriptions of abbreviations. S1: Adventure Bank sector in Sicilian waters, S2 Maltese Bank sector in Sicilian waters, G1: Thermaikos Gulf, G2: Thracian Sea.

**Fig 7:**
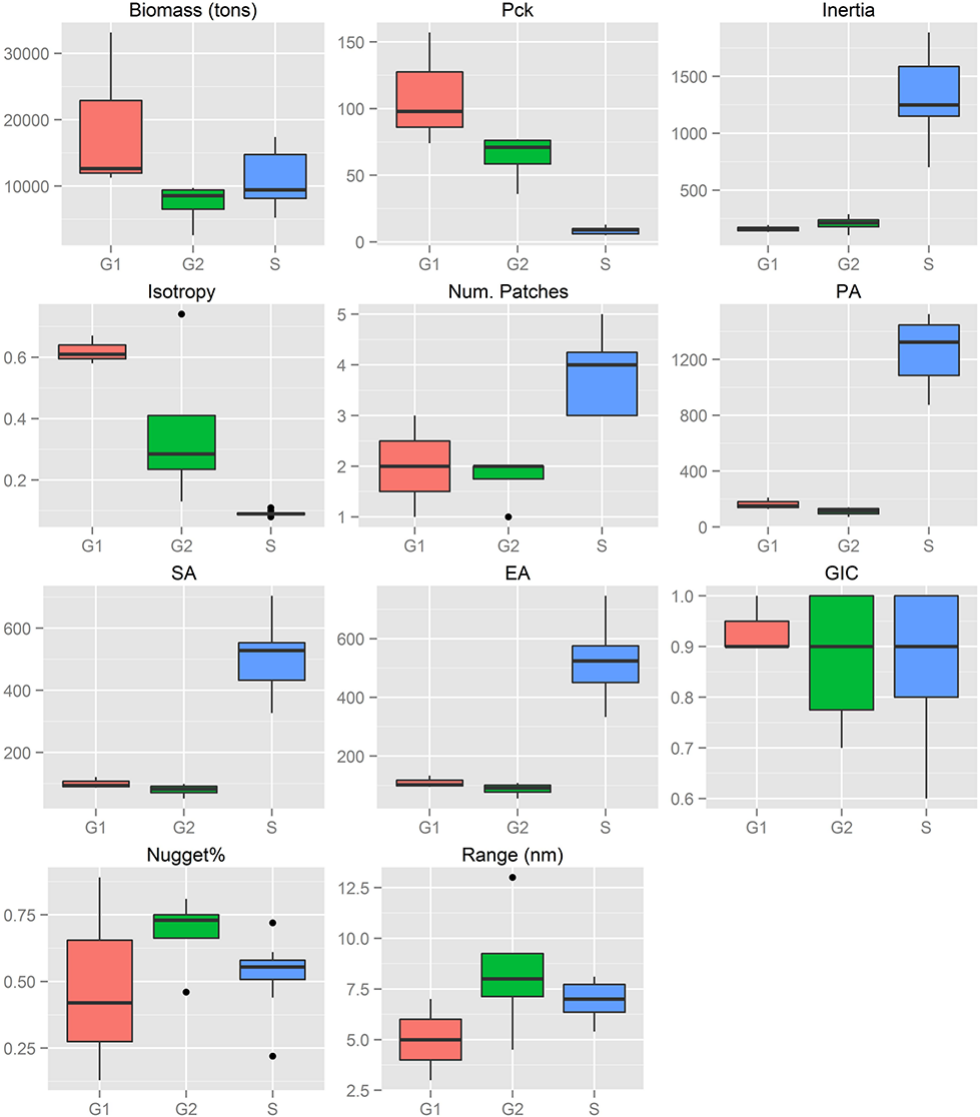
Sardine: Box-plots of biomass (t), packing density (Pck) and spatial indicator values per sub-area. See Table 1 for descriptions of abbreviations. S1: Adventure Bank sector in Sicilian waters, S2 Maltese Bank sector in Sicilian waters, G1: Thermaikos Gulf, G2: Thracian Sea.

Marked differences in biomass levels among sub-areas were evidenced for both anchovy (Fig 6) and sardine (Fig 7). Pronounced differences were also observed in the Pck, which was almost 10 times higher in the North Aegean Sea compared to the Strait of Sicily for both species (Figs 6 to 7).

### Indicator variography results

The threshold to define aggregations representing the dominant part of the abundance (values >c80) varied greatly between species and areas. On average, the biomass of anchovy was six times higher in the Greek sub-areas than in the Strait of Sicily (Fig 6) while the biomass of sardine was more similar between the two ecosystems (Fig 7). However, the thresholds to define the dominant aggregations in the indicator variograms were ~50 times higher in the Greek sub-areas than in the Strait of Sicily (Table in S1 Table). This means that, at EDSU scale, anchovy and especially sardine are much more densely distributed in the Greek sub-areas than in the Strait of Sicily.

Anchovy spatial structures on the Maltese Bank (S2) and in the Thracian Sea (G2), as well as sardine in the Thermaikos Gulf (G1), presented the highest interannual variability in terms of nugget (Figs 6 and 7). Indicator variograms showed that anchovy formed, on average, bigger patches in the Strait of Sicily (autocorrelation range at 12.5 nmi in S1 and 10.9 nmi in S2) compared to the North Aegean Sea (6.5 nmi in G1 and 7.6 nmi in G2). For sardine, the lowest range was observed in G1 (5 nmi on average), while in G2 and S the average patch dimension was 7 and 8.4 nmi respectively (Figs 6 and 7). It should be noted that the specific topography of the Strait of Sicily (narrow continental shelf especially in the central part of the study area) causes anisotropy along the East-West direction in the spatial aggregations of small pelagic fish. Anisotropy was especially apparent for sardine, which was distributed in the central part of the continental shelf mainly (Fig 3). The presence of anisotropy might have introduced some bias in the estimation of the autocorrelation range and the nugget. However, because the directional variograms are often much noisier than the omnidirectional variograms, we decided to use the omnidirectional ones for our comparative analysis, for purposes of consistency with the variograms of the North Aegean Sea.

### Spatial indicators

Spatial indicators were computed per year, species and sub-area (Figs 6 to 7, data in S2 Table) and revealed differences in the spatial patterns of the two species. Concerning location, both species presented highly consistent CGs in Greek waters (Figs 4 and 5) and so did sardine in the Strait of Sicily (Fig 3). Specifically, the distance between CGs ranged from 0.7 nmi – 6 nmi in Thermaikos Gulf, 1.05 nmi to 14.27 nmi in the Thracian Sea and 1.3 nmi to 16 nmi in the Strait of Sicily. On the other hand, the location of anchovy in the Strait of Sicily was more variable between years (Fig 2, ranged from 0.228 nmi to 25 nmi). Both species presented a consistently higher occupation index, expressed as higher positive area (PA), in the Strait of Sicily compared to Greek waters (Figs 6 to 7). This was more pronounced in sardine, which presented almost nine times higher PA in the Strait of Sicily. In contrast to the occupation index, SA and EA (aggregation indices) for anchovy were on average ~200 to 250 nmi^2^ independently of study area (Fig 6). This was not the case for sardine in which SA and EA differed largely among areas, ranging from 100 nmi^2^ in Greek sub-areas to 500 nmi^2^ in the Strait of Sicily (Fig 7). Dispersion also differed between areas and species. In Sicilian waters, both species showed a marked anisotropic pattern, evidenced by the low values of the isotropy indicator (Figs 6 to 7). In Greek waters, anchovy was characterized by a more isotropic behaviour. The marked differences in Inertia values also revealed higher dispersion in Sicilian waters (being more pronounced in the case of sardine) compared to the North Aegean Sea (Figs 6 to 7). Patchiness showed common patterns independently of species and ecosystem. Both anchovy and sardine presented 3 to 5 major patches (Figs 6 and 7). Finally, in the North Aegean Sea, the high GIC values denote a high degree of overlap between anchovy and sardine populations in both sub-areas (>92%, Fig 6). This was not the case in the Strait of Sicily, where anchovy and sardine overlapped only occasionally in S1 (0.36 to 0.99) and were collocated more often in S2 (0.72 to 0.96).

### Principal Component analysis results

**Fig 8:**
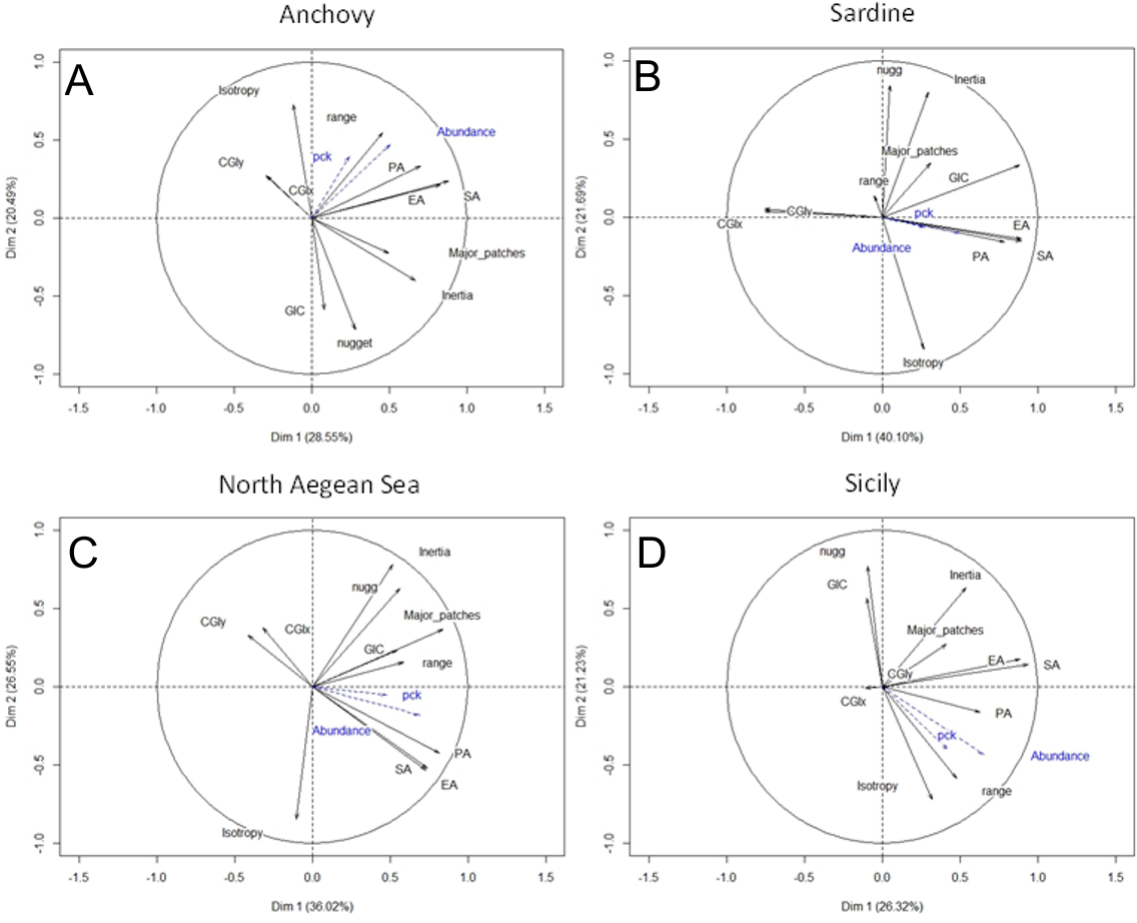
PCA biplots for (A) anchovy (B) sardine (C) North Aegean Sea and (D) Strait of Sicily. Active variables (spatial indicators) are shown in black. Descriptions of the spatial indicators are given in Table 1. Supplementary variables (biomass and packing density (Pck)) are shown in blue.

Principal Component Analysis allowed the visualization of correlations among a suite of active variables (spatial indicators) thus highlighting the relationships among them. The loadings of space occupation, aggregation and patchiness (number of major patches) in PC1 were highly positive for both species and areas (Fig 8, Tables 2 and 3). In the case of anchovy, the autocorrelation range (i.e. variogram range), nugget and dispersion (i.e. inertia) also had highly positive loadings in PC1 (Fig 8, Table 2). In the case of sardine, autocorrelation range, nugget and inertia were not significant in PC1 (Fig 8, Table 2). Furthermore, the CG in sardine exhibited high contrast (negative loading) with the remaining indicators in PC1 (Fig 8, Table 2). Looking at the PCA results by area (Fig 8, Table 3), the loadings of dispersion, spatial occupation, aggregation and patchiness in PC1 were highly positive in the Strait of Sicily. In the North Aegean Sea, the PC1 loading of overlap (GIC), rather than dispersion, was highly positive (Fig 8, Table 3).

**Table 2:** Principal Component loadings per species. Supplementary variables are shown in italics. Significantly correlated variables on each axis are marked with an (*) and shown in bold.

Species	Variable	PC1	PC2	PC3	PC4	PC5
**Anchovy**	CGIx	-0.29	0.27	0.89*	-0.06	0.13
	CGIy	-0.29	0.27	0.88*	-0.04	0.18
	Inertia	0.67*	-0.40	0.23	-0.21	-0.23
	Isotropy	-0.12	0.73*	-0.30	0.06	0.03
	Major_patches	0.50*	-0.22	-0.12	0.44*	0.61*
	PA	0.70*	0.33	0.17	0.46*	-0.03
	SA	0.88*	0.24	0.07	-0.37	0.00
	EA	0.83*	0.21	0.06	-0.48*	0.05
	GIC	0.08	-0.59*	0.30	0.28	-0.63*
	range	0.46*	0.55*	0.13	0.52*	-0.28
	nugget	0.28	-0.71*	0.25	0.13	0.40
	***Biomass***	*0*.51*	*0*.47*	*0*.*00*	*0*.*05*	*0*.*32*
	***Pck***	*0*.*24*	*0*.*40*	*-0*.*09*	*-0*.*18*	*0*.*34*
**Sardine**	CGIx	-0.76*	0.05	0.58*	0.18	0.22
	CGIy	-0.75*	0.04	0.60*	0.16	0.20
	Inertia	0.30	0.80*	-0.06	0.15	0.41
	Isotropy	0.27	-0.84*	0.30	0.20	-0.08
	Major_patches	0.88*	0.33	-0.16	0.08	0.10
	PA	0.79*	-0.16	0.28	0.01	-0.20
	SA	0.89*	-0.15	0.30	0.15	0.23
	EA	0.90*	-0.14	0.31	0.14	0.22
	GIC	0.31	0.35	0.59*	-0.43	-0.39
	range	-0.05	0.14	-0.12	0.90*	-0.37
	nugget	0.05	0.84*	0.29	0.09	-0.27
	***Biomass***	*0*.*50*	*-0*.*10*	*0*.62*	*0*.*37*	*-0*.*22*
	***Pck***	*0*.*28*	*-0*.*06*	*0*.65*	*0*.*39*	*-0*.*20*

**Table 3:** Principal Component loadings per area. Supplementary variables are shown in italics. Significantly correlated variables on each axis are marked with an (*) and shown in bold.

Area	Variable	PC1	PC2	PC3	PC4	PC5
**Sicily**	CGIx	0.04	0.00	-0.99*	-0.05	0.06
	CGIy	-0.11	-0.01	0.99*	0.06	0.00
	Inertia	0.54*	0.63*	-0.04	0.08	-0.27
	Isotropy	0.32	-0.72*	-0.05	-0.12	0.18
	Major_patches	0.41	0.27	0.05	-0.31	0.66*
	PA	0.63*	-0.16	0.04	0.54*	0.36
	SA	0.94*	0.14	0.06	-0.16	-0.18
	EA	0.89*	0.18	0.08	-0.27	-0.25
	GIC	-0.10	0.57*	-0.15	0.66*	-0.10
	range	0.48*	-0.59*	0.00	0.52*	0.00
	nugget	-0.09	0.77*	0.07	0.06	0.40
	***Biomass***	*0*.65*	*-0*.44*	*0*.*10*	*-0*.*10*	*0*.*22*
	***Pck***	*0*.42*	*-0*.*40*	*0*.*11*	*-0*.*38*	*0*.*03*
**North Aegean Sea**	CGIx	-0.32	0.38	0.86*	0.04	0.08
	CGIy	-0.41	0.33	0.83*	0.08	0.16
	Inertia	0.52	0.78*	0.20	-0.11	-0.18
	Isotropy	-0.10	-0.85*	0.12	-0.10	0.42
	Major_patches	0.84*	0.37	-0.24	-0.11	0.06
	PA	0.82*	-0.42	0.31	0.01	-0.11
	SA	0.74*	-0.52	0.39	0.05	-0.14
	EA	0.73*	-0.53*	0.38	0.07	-0.14
	GIC	0.59*	0.16	-0.18	0.60*	0.42
	range	0.55*	0.23	0.04	-0.71*	0.33
	nugget	0.57*	0.63*	-0.01	0.22	0.09
	***Biomass***	*0*.69*	*-0*.*18*	*0*.*19*	*0*.*30*	*0*.*20*
	***Pck***	*0*.*48*	*-0*.*05*	*0*.*09*	*0*.*41*	*0*.*39*

Regarding PC2, sardine exhibited highly positive loadings for nugget and inertia that are in contrast with isotropy (Fig 8, Table 2). Anchovy exhibited highly positive PC2 loadings for nugget, inertia and overlap (GIC), which are also in contrast with isotropy (Fig 8, Table 2). In the North Aegean Sea, nugget and inertia presented highly positive loadings while the loading for isotropy was negative (Fig 8, Table 3). In Sicily, nugget, inertia and GIC were in contrast with the range and isotropy in PC2 (Fig 8, Table 3).

The position of the centre of gravity (CGIx, CGIy) was positive along PC3 in all species and areas (Tables 2 and 3).

For anchovy, in all PCAs, standardized biomass was significantly and positively correlated to PC1 (Fig 8, Tables 2 and 3) whereas the same was true for Pck in the Strait of Sicily (Table 3). Standardized biomass was also positively correlated with PC2 for anchovy and negatively correlated in the Strait of Sicily (Fig 8, Tables 2 and 3). For sardine, standardized biomass and Pck were significantly positive but only along the PC3 axis (Table 2).

### Multiple regression

**Fig 9:**
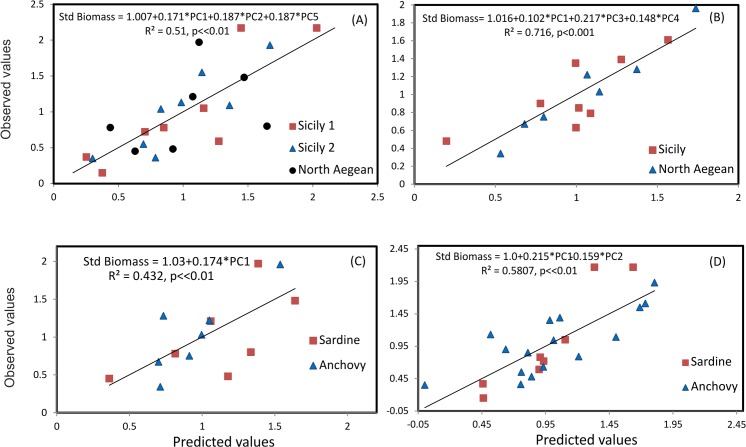
Observed vs predicted values for multiple regression models of standardized fish biomass on PCA axes scores, per species and area: a) pooled anchovy data from both areas; b) pooled sardine data from both areas; c) pooled anchovy and sardine data from the North Aegean Sea and d) pooled anchovy and sardine data from the Strait of Sicily. The estimated equation in each case is also shown.

Multiple regression analysis was applied to relate the standardized biomass of anchovy and sardine with the PCA scores of the first five PCs (data in S3 Table and S4 Table), i.e. the most important PCs accounting for > 85% of the variance. Results showed that for anchovy, biomass values were positively related to occupation, aggregation, patchiness and dispersion as well as negatively related to nugget and collocation with sardine (Fig 9A). Similarly, for sardine, biomass was positively related to occupation, aggregation, spatial patches and the collocation with anchovy (Fig 9B). In both areas, an increase in biomass resulted into an increase in occupation, aggregation, nugget and the formation of more and bigger major patches (Fig 9C and 9D). In the North Aegean Sea, the increase in biomass resulted in an increase in the overlap between anchovy and sardine populations (Fig 9C). In contrast, in the Strait of Sicily, an increase in biomass was associated with the reduction of the overlap between anchovy and sardine as well as a decrease in nugget (Fig 9D).

## Discussion

This study focused on the aggregation patterns of anchovy and sardine populations in two different ecosystems: an upwelling area, the Strait of Sicily and a continental shelf area, the North Aegean Sea. Biomass was generally higher in the North Aegean Sea compared to the Strait of Sicily, and in most cases, higher for anchovy than sardine. In the Strait of Sicily, the biomass of both species displayed high interannual variability; however, the spatial distribution of their populations was quite similar between years, as shown by the centres of gravity and the location of the main spatial patches.

In the North Aegean Sea, the biomass of anchovy and sardine exhibited high interannual variability as well. The centres of gravity and the location of the main spatial patches were similar between years in the Thermaikos Gulf (G1), but much more variable in the Thracian Sea (G2), especially for sardine. The consistency of the population centres in the Strait of Sicily and the Thermaikos Gulf (G1) indicates that the locations of the main small pelagic fish concentrations might be associated with specific environmental features, which do not change much between years, such as the rivers outflow in the Thermaikos Gulf and the permanent upwelling over the Adventure Bank in the Strait of Sicily. The variability observed in the Thracian Sea can be attributed to the temporal changes in the position of the anticyclonic system generated over the Samothraki plateau due to the advection of Black Sea Water [29],[38]. Similar stability in the spatial structures and the centres of gravity was observed for anchovy and sardine populations in the Gulf of Lion (Western Mediterranean Sea) [9]. In this case, anchovies consistently occupied the centre of the continental shelf, while sardines were distributed in closer proximity to the coast [9].

In the Strait of Sicily, anchovy patches (as defined by the indicator autocorrelation range) tend to be bigger and more variable in size compared to the ones of sardine whereas both species form similar-sized patches in the North Aegean Sea. Sardine generally presented smaller patches compared to anchovy independently of biomass. This implies that the size of patches is mostly area-(e.g. related to local environmental conditions) and species-specific rather than biomass-dependent. It was noted that biomass level has no effect on the autocorrelation range of the high density indicator variograms, a fact that has also been reported for anchovy and sardine in the southern Africa upwelling area [4].

In the Strait of Sicily, both species presented consistently higher occupation values (area of presence) compared to the North Aegean Sea. This was more pronounced for sardine. As opposed to occupation, anchovy exhibited similar aggregation patterns (the way in which a given population is distributed in its area of presence) in both ecosystems. On the other hand, the spreading area of sardine was more extended in the Strait of Sicily compared to the North Aegean Sea. Dispersion (inertia and isotropy) varied between ecosystems but not between species. Thus, occupation appears to be habitat-dependent primarily whereas the aggregation patterns may be species-specific and biomass-related. In the Strait of Sicily, both species exhibited more elongated populations than in the North Aegean Sea (lower isotropy values). This was largely determined by the narrow continental shelf in the central part of the Strait of Sicily, which imposes the formation of a higher number of patches along the West-East direction rather than the North-South.

The estimated degree of overlap between anchovy and sardine populations could be related to interspecific competition. In the Strait of Sicily, the low GIC values denote reduced overlap between anchovy and sardine. Here, the suitable habitat for small pelagics is limited along the narrow band of the south coast of Sicily and over the Adventure and Maltese Banks. Studies on habitat suitability modelling, using acoustic survey data from the Aegean Sea and the Strait of Sicily along with satellite environmental information [39], [40], has shown common environmental preferences for the two species during summer, e.g. <100 m bottom depth and SST<22°C. Thus, both species tend to separate their population centres (sardine is distributed in the narrow band of the central sector of the Strait of Sicily mainly whereas anchovy is distributed over the banks), probably to reduce inter-specific competition and exploit most of the available resources. A similar pattern has been observed in large upwelling ecosystems such as Benguela [4] where anchovy and sardine separate their spatial niche, sardine being mainly distributed inshore. Such interspecific spatial segregation was not observed in the North Aegean Sea, where the populations of the two species largely overlapped (high GIC values). In this continental shelf area, local environmental characteristics generate strongly localized food resources associated with freshwater sources and specific hydrographic features, like the Samothraki gyre [39], [40], [41], [42].

The difference between the thresholds of the indicator variograms (used to define those aggregations representing most part of the abundance) indicated that at the 1 nmi scale (the EDSU), anchovy and especially sardine were much more densely distributed in the Greek sub-areas than in the Strait of Sicily. Dense populations that overlap and exploit localized food resources can make anchovy and sardine adopt other mechanisms in order to reduce competition. This might involve a food selection strategy although recent studies indicate that food partitioning between anchovy and sardine is less pronounced in the Mediterranean than in upwelling areas [43], [44]. Habitat suitability plays a major role in determining the spatial distribution of fish and the intrinsic difficulty in determining this property represents one of the major drawbacks in assessing the relative importance of density-dependent and independent factors. According to [9] our perception of the relative importance of density-dependent versus density-independent (environmental) processes on the spatial distributions of small pelagic fish largely depends on the spatial scale of the analysis.

At the spatial scale of this study, a large set of spatial indicators was analysed following a multivariate approach (PCA analysis) and strong correlations emerged between biomass and the first principal component, with highly positive loadings for occupation, aggregation and patchiness, independently of species and ecosystems. The latter relationships imply that when biomass increases, the populations of both species exhibit an expansion of the occupied space and concurrently form more major patches. This is in conformity with the dynamic D4 scenario suggested by [17], according to which the area of fish presence changes with population abundance, and the basin model of [45] (MacCall 1990) for anchovies, where density changes with abundance as a result of relationships between habitat suitability and local density. This spatial behaviour is known for large upwelling ecosystems (e.g. California Current, Peru-Humboldt Current), where small pelagic fish often expand their distribution range with increasing biomass and limit their distribution to specific locations, acting as refugia, when biomass decreases ([46] and references there in). In these ecosystems, the spatial distribution of the anchovy population often appears to be inversely related to that of the sardine population while changes in population size are associated with major geographical changes ([46] and references there in).

The principal component analysis of the indicators calculated in this study also showed that both anchovy and sardine presented strong correlation but opposite patterns between biomass and certain principal components in which the loading of overlapping (GIC values) was highly significant (negative and positive, respectively). In other words, when anchovy biomass increases in a given area, its population tends to expand in space and decrease its overlap with sardine. On the other hand, when sardine biomass increases, its population expands and its spatial overlap with anchovy increases. This contrasting pattern was attributed to the location of the respective major patches and the CG, combined with the specific occupation patterns of the two species in the study areas. In the Strait of Sicily, anchovy was mainly distributed over the Adventure and Maltese Banks. An increase in its biomass resulted in population expansion over the Banks and, subsequently, spatial overlapping with the sardine population decreased as this species was located in the narrow, central band of the Strait of Sicily mainly. As sardine biomass increased, the sardine population expanded over the Maltese Bank and overlapping with anchovy increased. In the North Aegean Sea, the populations of the two species largely overlapped like two concentric circles but with anchovy being distributed over a larger spreading area. Thus, an increase in sardine biomass resulted in expansion of the distribution area and, subsequently, an increase of the spatial overlap with the anchovy population.

The multivariate analysis revealed additional differences between the two species. Sardine exhibited a positive correlation between biomass and the autocorrelation range along the fourth principal component. Hence, an increase in sardine biomass is associated with larger high density patches. In anchovy, a positive correlation existed between biomass and the first and second principal components in which the loadings of the autocorrelation range (size of patches), isotropy and inertia were highly positive. Furthermore, a negative correlation existed between biomass and nugget along the second principal component. All these correlations suggest that an increase in anchovy biomass is accompanied by larger, but also better structured high density patches and more disperse and isotropic aggregations that overlap to a lesser degree with sardine.

The multivariate analysis also revealed similarities and differences between the two areas. A positive correlation between biomass and isotropy was found along PC2 in both areas. This means that an increase in biomass is related to more isotropic aggregations in both areas. However, in the North Aegean Sea, a positive correlation between biomass and overlap (GIC) along PC1 was found. In the Strait of Sicily, on the other hand, a positive correlation between biomass and dispersion was found along PC1. This means that a biomass increase in the North Aegean Sea could result in increased overlapping between the populations of the two species whereas in the Strait of Sicily a biomass increase would most likely result in more dispersed populations. Moreover, in the North Aegean Sea, a positive correlation between biomass and nugget was found along PC2 whereas a negative correlation with nugget was found in the Strait of Sicily. Thus, an increase in biomass was associated with less well-structured aggregations in the North Aegean Sea whereas the opposite was observed in the Strait of Sicily.

Except for the relationship between abundance and geographic distribution area, which has already been shown in several ecosystems (e.g. California, South Africa, Peru and Japan) [20], to our knowledge, no other clear relationships between abundance or biomass and other spatial patterns (e.g. population patchiness, size of high-density patches, overlap with another species, isotropy of the aggregations) have ever been demonstrated for small pelagic fish. The multivariate approach followed in this study showed that biomass is significantly related to a suite of rather than single spatial indicators, which were summarized in specific principal components. The study of similar relationships for other pelagic fishes in different ecosystems could improve our knowledge of the spatial patterns of fish and help in assessing the potential use of spatial indices as auxiliary stock monitoring indicators. The catchability of small pelagic fish depends on how well fishers perceive the spatial patterns of fish and how successfully they identify high density fish patches [5]. Low abundance levels associated with the shrinkage of the distribution area of the fish population can easily result in rapid overfishing and render the stock susceptible to population collapse [47]. This is highly relevant for sardine populations in the North Aegean Sea, which exhibit smaller spreading area values (almost half) compared to anchovy. Based on our findings, a decrease in sardine biomass would result in further reduction of the spreading area and the formation of smaller patches, which can make sardine more vulnerable to fishing pressure. Taking into account that the sardine populations in the North Aegean Sea are currently overexploited [25], [26], changes in spatial patterns are of special interest from a management perspective. Detailed knowledge of the spatial patterns of fish and how they respond to changes in population abundance can greatly assist in the establishment of effective marine protected areas [22].

## Supporting Information

S1 Table Indicator variogram descriptors for anchovy and sardine. (DOCX)

S2 Table Estimated spatial indicators for anchovy and sardine. (DOCX)

S3 Table PCA scores per species. (DOCX)

S4 Table PCA scores per area. (DOCX)
