# Supplementary material for: Interannual Changes in Biomass Affect the Spatial Aggregations of Anchovy and Sardine as Evidenced by Geostatistical and Spatial Indicators
Source: PLoS One. 2015 Aug 27;10(8):e0135808. doi: 10.1371/journal.pone.0135808 (PMC4551744; doi:10.1371/journal.pone.0135808)
Supplement: S1 Table — (DOCX) [file pone.0135808.s001.docx]

**S1 Table. :** Summary table reporting the indicator variogram descriptors for anchovy and sardine in each considered area (S1: Adventure Bank sector in Sicily waters, S2: Maltese Bank sector in Sicily waters, S: Sicily waters, G1: Thermaikos Gulf, G2: Thracian Sea)

|  | **Area** | **Year** | **Biomass (tons)** | **Threshold** | **Nugget (%)** | **Range (nmi)** |
| --- | --- | --- | --- | --- | --- | --- |
| **Anchovy** | **S1** | 2002 | 601 | 1 | 0.5 | 4.3 |
|  | **S1** | 2003 | 3023 | 7 | 0.3 | 14.6 |
|  | **S1** | 2005 | 8449 | 19 | 0.4 | 11.9 |
|  | **S1** | 2006 | 4085 | 8 | 0.4 | 11.9 |
|  | **S1** | 2007 | 2279 | 4 | 0.5 | 10.8 |
|  | **S1** | 2008 | 1427 | 3 | 0.6 | 10.8 |
|  | **S1** | 2009 | 2806 | 4 | 0.6 | 17.3 |
|  | **S1** | 2010 | 8417 | 14 | 0.7 | 18.9 |
|  | **S2** | 2002 | 8564 | 9 | 0.3 | 13.5 |
|  | **S2** | 2003 | 4612 | 11 | 0.4 | 14.6 |
|  | **S2** | 2005 | 4824 | 5 | 0.5 | 11.9 |
|  | **S2** | 2006 | 1620 | 3 | 0.5 | 9.1 |
|  | **S2** | 2007 | 5026 | 11 | 0.8 | 6.4 |
|  | **S2** | 2008 | 2426 | 8 | 0.4 | 4.3 |
|  | **S2** | 2009 | 1556 | 5 | 0.85 | 3.2 |
|  | **S2** | 2010 | 6885 | 20 | 0.2 | 24.3 |
|  | **G1** | 2004 | 25142 | 500 | 0.6 | 4.5 |
|  | **G1** | 2005 | 36517 | 300 | 0.6 | 7 |
|  | **G1** | 2006 | 14454 | 275 | 0.5 | 8 |
|  | **G2** | 2004 | 44876 | 300 | 0.1 | 4 |
|  | **G2** | 2005 | 22696 | 100 | 0.5 | 9 |
|  | **G2** | 2006 | 9992 | 75 | 0.4 | 12 |
|  | **G2** | 2008 | 17398 | 2000 | 0.8 | 6 |
| **Sardine** | **S** | 2002 | 5230 | 7 | 0.22 | 8.1 |
|  | **S** | 2003 | 9176 | 6 | 0.57 | 5.4 |
|  | **S** | 2005 | 17403 | 10 | 0.44 | 8.1 |
|  | **S** | 2006 | 8575 | 6 | 0.56 | 7 |
|  | **S** | 2007 | 9704 | 11 | 0.61 | 7 |
|  | **S** | 2008 | 15033 | 10 | 0.72 | 6.5 |
|  | **S** | 2009 | 6859 | 5 | 0.53 | 5.9 |
|  | **S** | 2010 | 14651 | 16 | 0.55 | 7.6 |
|  | **G1** | 2004 | 10581 | 9800 | 0.13 | 5 |
|  | **G1** | 2005 | 11254 | 2400 | 0.89 | 3 |
|  | **G1** | 2006 | 12643 | 1770 | 0.42 | 7 |
|  | **G2** | 2004 | 33128 | 198 | 0.81 | 8 |
|  | **G2** | 2005 | 8700 | 1000 | 0.73 | 8 |
|  | **G2** | 2006 | 2594 | 2100 | 0.46 | 13 |
|  | **G2** | 2008 | 7821 | 13500 |  |  |

**S2 Table:** Summary table reporting the estimated spatial indicators for anchovy and sardine in each considered area (S1: Adventure Bank sector in Sicily waters, S2: Maltese Bank sector in Sicily waters, S: Sicily waters, G1: Thermaikos Gulf, G2: Thracian Sea)

|  | **Area** | **Year** | PA | SA | EA | GIC | Inertia | Isotropy | Major_patches |
| --- | --- | --- | --- | --- | --- | --- | --- | --- | --- |
| **Anchovy** | **S1** | 2002 | 0.77 | 0.8 | 0.78 | 1.23 | 1.06 | 1.06 | 2 |
|  | **S1** | 2003 | 0.69 | 0.89 | 0.94 | 0.79 | 0.82 | 0.89 | 2 |
|  | **S1** | 2005 | 0.71 | 1.1 | 1.21 | 0.55 | 0.84 | 1.25 | 2 |
|  | **S1** | 2006 | 1.24 | 1.06 | 1.01 | 0.9 | 0.59 | 0.94 | 2 |
|  | **S1** | 2007 | 0.91 | 1.04 | 1.08 | 0.89 | 0.84 | 0.91 | 3 |
|  | **S1** | 2008 | 0.95 | 0.86 | 0.81 | 1.5 | 1.4 | 0.88 | 2 |
|  | **S1** | 2009 | 1.22 | 1.11 | 1.07 | 1.51 | 1.23 | 0.86 | 2 |
|  | **S1** | 2010 | 1.49 | 1.14 | 1.11 | 0.63 | 1.23 | 1.21 | 3 |
|  | **S2** | 2002 | 1.33 | 1.38 | 1.38 | 0.83 | 1.08 | 1.09 | 2 |
|  | **S2** | 2003 | 0.98 | 0.94 | 1.01 | 1.11 | 0.76 | 0.9 | 2 |
|  | **S2** | 2005 | 0.97 | 1.53 | 1.62 | 1.09 | 1.93 | 0.98 | 2 |
|  | **S2** | 2006 | 0.92 | 0.98 | 1.01 | 1.02 | 0.78 | 0.83 | 2 |
|  | **S2** | 2007 | 0.88 | 0.85 | 0.86 | 0.93 | 0.72 | 0.89 | 3 |
|  | **S2** | 2008 | 0.77 | 0.74 | 0.67 | 0.88 | 0.66 | 1.25 | 2 |
|  | **S2** | 2009 | 0.81 | 0.75 | 0.73 | 1.05 | 1.35 | 0.74 | 2 |
|  | **S2** | 2010 | 1.32 | 0.83 | 0.71 | 1.08 | 0.72 | 1.32 | 2 |
|  | **G1** | 2004 | 0.95 | 0.89 | 0.9 | 0.96 | 0.73 | 1.23 | 2 |
|  | **G1** | 2005 | 0.93 | 0.92 | 0.93 | 1.05 | 0.82 | 0.92 | 2 |
|  | **G1** | 2006 | 1.25 | 1.16 | 1.15 | 1.09 | 1.16 | 0.78 | 3 |
|  | **G2** | 2004 | 0.71 | 0.75 | 0.76 | 0.75 | 0.45 | 1.09 | 1 |
|  | **G2** | 2005 | 0.75 | 0.59 | 0.57 | 1.13 | 1.02 | 0.93 | 2 |
|  | **G2** | 2006 | 1.29 | 1.18 | 1.18 | 0.88 | 1.11 | 1.15 | 2 |
|  | **G2** | 2008 | 1.13 | 1.17 | 1.19 | 1.12 | 1.17 | 0.84 | 2 |
| **Sardine** | **S** | 2002 | 0.69 | 0.65 | 0.64 | 0.71 | 0.53 | 1 | 2 |
|  | **S** | 2003 | 0.89 | 1.08 | 1.08 | 1.13 | 1.14 | 0.84 | 5 |
|  | **S** | 2005 | 1.05 | 1.39 | 1.42 | 0.91 | 1.33 | 1.19 | 6 |
|  | **S** | 2006 | 1.21 | 1.08 | 1.01 | 0.88 | 0.91 | 1.06 | 3 |
|  | **S** | 2007 | 0.78 | 0.81 | 0.86 | 1.15 | 0.96 | 0.95 | 3 |
|  | **S** | 2008 | 1.14 | 1.12 | 1.14 | 1.07 | 0.87 | 0.98 | 5 |
|  | **S** | 2009 | 1.06 | 1 | 1 | 1.04 | 1.41 | 0.92 | 4 |
|  | **S** | 2010 | 1.19 | 0.87 | 0.86 | 1.11 | 0.83 | 1.05 | 3 |
|  | **G1** | 2004 | 0.8 | 0.79 | 0.78 | 0.96 | 0.67 | 1.06 | 1 |
|  | **G1** | 2005 | 0.68 | 0.73 | 0.73 | 1.05 | 0.77 | 1.01 | 2 |
|  | **G1** | 2006 | 1.11 | 1.01 | 1.02 | 1.09 | 0.97 | 1.18 | 3 |
|  | **G2** | 2004 | 0.5 | 0.53 | 0.51 | 0.75 | 1.41 | 0.33 | 2 |
|  | **G2** | 2005 | 0.72 | 0.79 | 0.78 | 1.13 | 0.99 | 0.75 | 2 |
|  | **G2** | 2006 | 0.9 | 1.01 | 1.01 | 0.88 | 0.51 | 1.87 | 1 |
|  | **G2** | 2008 | 0.99 | 0.91 | 0.91 | 1.12 | 1.09 | 0.67 | 2 |

**S3 Table.** PCA scores per species. (S1: Adventure Bank sector in Sicily waters, S2 Maltese Bank sector in Sicily waters, S: Sicily waters, G1: Thermaikos Gulf, G2 Thracian Sea)

|  | **Area** | **Stand Biomass** | **PC1** | **PC2** | **PC3** | **PC4** | **PC5** |
| --- | --- | --- | --- | --- | --- | --- | --- |
| **Anchovy** | **S1** | 0.1500 | -1.3807 | -1.4917 | -0.9616 | -0.3168 | -0.6308 |
|  | **S1** | 0.7800 | -1.0175 | 0.3066 | -1.2989 | -0.5689 | -0.2207 |
|  | **S1** | 2.1700 | -0.1375 | 1.5547 | -1.7824 | -1.6492 | 0.9389 |
|  | **S1** | 1.0500 | 0.2098 | 0.5434 | -0.9113 | 0.1994 | 0.0739 |
|  | **S1** | 0.5900 | 0.6881 | -0.6642 | -1.0401 | 0.1961 | 1.5014 |
|  | **S1** | 0.3700 | 0.0390 | -2.3534 | 0.0368 | 0.5270 | -1.7473 |
|  | **S1** | 0.7200 | 1.7008 | -1.2593 | 0.3599 | 0.8187 | -1.9498 |
|  | **S1** | 2.1700 | 2.7775 | 1.1165 | -0.8726 | 1.4226 | 1.8515 |
|  | **S2** | 1.9300 | 2.3998 | 1.8295 | -0.8675 | -0.9177 | -0.4943 |
|  | **S2** | 1.0400 | -0.0304 | -0.0541 | -0.7433 | 0.4416 | -0.8970 |
|  | **S2** | 1.0900 | 3.7870 | -0.3614 | -0.0303 | -2.5371 | -1.2524 |
|  | **S2** | 0.3600 | -0.2224 | -0.8216 | -0.7395 | -0.3545 | -0.1587 |
|  | **S2** | 1.1300 | -0.3354 | -1.7348 | -1.0625 | 0.7871 | 1.9731 |
|  | **S2** | 0.5500 | -2.3974 | 0.1949 | -1.9171 | -0.1165 | 0.3276 |
|  | **S2** | 0.3500 | -0.7300 | -3.5830 | -0.1723 | -0.3295 | 0.4933 |
|  | **S2** | 1.5500 | -0.1432 | 2.5001 | -1.2154 | 2.7705 | -1.6746 |
|  | **G1** | 1.2100 | -1.4364 | 0.8919 | 1.0909 | -0.0357 | 0.7960 |
|  | **G1** | 0.4800 | -0.8637 | 0.2195 | 1.6908 | 0.2210 | 0.1108 |
|  | **G1** | 1.4800 | 1.8184 | 0.0219 | 2.1283 | 0.8697 | 0.8125 |
|  | **G2** | 0.4500 | -4.0887 | 2.1954 | 0.9357 | -1.6444 | -0.4930 |
|  | **G2** | 0.7800 | -2.6066 | -0.4345 | 2.1242 | 0.9314 | -0.2287 |
|  | **G2** | 0.8000 | 1.0728 | 2.2579 | 2.2349 | 0.0320 | 0.1806 |
|  | **G2** | 1.9700 | 0.8968 | -0.8744 | 3.0133 | -0.7468 | 0.6878 |
| **Sardine** | **S** | 0.4800 | -1.7091 | -2.1344 | -3.0968 | 0.2021 | -0.3653 |
|  | **S** | 0.8500 | 2.0817 | 0.9622 | -0.2765 | -1.0400 | 0.3762 |
|  | **S** | 1.6100 | 3.9516 | -0.1805 | -0.3140 | 1.4358 | 1.6517 |
|  | **S** | 0.7900 | 1.6208 | -0.6525 | -0.5964 | 0.2347 | -0.2082 |
|  | **S** | 0.9000 | 0.2651 | 0.6290 | -0.6418 | -0.8493 | -1.0718 |
|  | **S** | 1.3900 | 2.6008 | 0.4675 | 0.1996 | -0.3293 | -0.5845 |
|  | **S** | 0.6300 | 1.8391 | 0.9111 | -0.5711 | -0.5585 | 0.6024 |
|  | **S** | 1.3500 | 1.0408 | -0.2481 | -0.2639 | -0.4733 | -1.5860 |
|  | **G1** | 0.6700 | -2.1040 | -1.9710 | 0.1712 | -1.0631 | 0.7301 |
|  | **G1** | 0.7500 | -2.0518 | 0.2065 | 0.7067 | -1.0908 | -0.1454 |
|  | **G1** | 1.9600 | 0.3302 | 0.4018 | 1.6617 | 2.1954 | -1.1304 |
|  | **G2** | 0.3400 | -3.8115 | 2.8875 | -1.3858 | 1.4015 | 0.8915 |
|  | **G2** | 1.0300 | -1.9865 | 1.3678 | 1.0697 | 0.6535 | -0.6072 |
|  | **G2** | 1.2800 | -1.1318 | -3.6187 | 1.5695 | 0.8868 | 0.4794 |
|  | **G2** | 1.2200 | -0.9351 | 0.9717 | 1.7678 | -1.6054 | 0.9673 |

**S4 Table.** PCA scores per area.

| **Area** | **Species** | **Stand Biomass** | **PC1** | **PC2** | **PC3** | **PC4** | **PC5** |
| --- | --- | --- | --- | --- | --- | --- | --- |
| **Sicily** | anchovy | 0.1500 | -2.0064 | 0.6676 | 0.8441 | -0.0860 | -0.5918 |
|  | anchovy | 0.7800 | -1.2944 | -1.1707 | 2.0595 | -0.6109 | -1.3933 |
|  | anchovy | 2.1700 | 0.2116 | -1.8069 | 2.3312 | -2.0642 | -0.9225 |
|  | anchovy | 1.0500 | -0.1576 | -0.8659 | 1.8739 | 0.4136 | -0.0278 |
|  | anchovy | 0.5900 | -0.2767 | 0.2530 | 1.8548 | -0.6589 | -0.0457 |
|  | anchovy | 0.3700 | -1.1225 | 1.8802 | 0.9803 | 1.8169 | -0.7724 |
|  | anchovy | 0.7200 | 0.6940 | 1.3436 | 1.6216 | 2.6362 | -0.7160 |
|  | anchovy | 2.1700 | 2.2483 | -0.9649 | 2.2282 | 0.5563 | 1.3635 |
|  | anchovy | 1.9300 | 2.8077 | -1.2650 | -1.5973 | -0.1328 | -0.9694 |
|  | anchovy | 1.0400 | -0.3365 | -0.5352 | -1.8936 | 0.7252 | -0.5470 |
|  | anchovy | 1.0900 | 3.6042 | 1.7088 | -1.8592 | -0.4690 | -2.4775 |
|  | anchovy | 0.3600 | -0.8270 | 0.4156 | -0.9501 | -0.1281 | -0.5263 |
|  | anchovy | 1.1300 | -1.5334 | 0.9471 | -1.1973 | -0.7548 | 1.0181 |
|  | anchovy | 0.5500 | -2.2068 | -1.4740 | -2.1047 | -1.2603 | 0.2089 |
|  | anchovy | 0.3500 | -2.2952 | 3.1624 | -1.3068 | -0.1883 | 0.2790 |
|  | anchovy | 1.5500 | 0.4587 | -3.7102 | -1.9122 | 2.4942 | 0.5814 |
|  | sardine | 0.4800 | -2.5447 | -2.4299 | -0.3640 | -0.6639 | -0.4421 |
|  | sardine | 0.8500 | 0.4043 | 1.7294 | 0.2670 | -0.6863 | 0.7439 |
|  | sardine | 1.6100 | 3.4984 | 0.1202 | -0.2772 | -1.5582 | 1.0552 |
|  | sardine | 0.7900 | 0.6595 | -0.4290 | -0.0260 | -0.0232 | 0.7278 |
|  | sardine | 0.9000 | -1.2512 | 0.5842 | -0.6031 | 0.1486 | 0.2297 |
|  | sardine | 1.3900 | 1.0666 | 1.0480 | 0.0267 | -0.3387 | 1.8674 |
|  | sardine | 0.6300 | 0.5596 | 1.1760 | -0.0314 | -0.1044 | 0.3981 |
|  | sardine | 1.3500 | -0.3603 | -0.3846 | 0.0357 | 0.9368 | 0.9589 |
| **Greece** | anchovy | 1.2100 | 0.1703 | -1.2357 | -1.4124 | 0.2747 | -0.2900 |
|  | anchovy | 0.4800 | 0.8510 | -0.5275 | -1.4260 | -0.1258 | -0.0297 |
|  | anchovy | 1.4800 | 3.5076 | -0.4627 | -0.7042 | -0.4858 | -0.7476 |
|  | anchovy | 0.4500 | -3.8433 | -1.5595 | 0.6044 | -0.2841 | -0.8084 |
|  | anchovy | 0.7800 | -1.2358 | 1.8642 | 0.0047 | 0.2128 | 1.4658 |
|  | anchovy | 0.8000 | 1.7550 | -0.8237 | 2.4678 | -1.4987 | -0.3101 |
|  | anchovy | 1.9700 | 2.0462 | 0.5009 | 1.9878 | 1.6663 | -0.4696 |
|  | sardine | 0.6700 | -1.8957 | -1.5970 | -1.4562 | 0.2242 | -0.5692 |
|  | sardine | 0.7500 | -0.6744 | 0.1594 | -2.1214 | 0.8705 | 0.1839 |
|  | sardine | 1.9600 | 2.9200 | -0.3708 | -1.1897 | -0.9300 | 0.8376 |
|  | sardine | 0.3400 | -1.8260 | 4.0953 | -0.0336 | -1.6265 | -0.8896 |
|  | sardine | 1.0300 | -0.1914 | 1.8008 | 0.4077 | 0.2715 | 1.0914 |
|  | sardine | 1.2800 | -1.6995 | -2.8711 | 1.7513 | -0.4402 | 1.0859 |
|  | sardine | 1.2200 | 0.1161 | 1.0274 | 1.1200 | 1.8710 | -0.5504 |
