# Supplementary material for: Interannual Changes in Biomass Affect the Spatial Aggregations of Anchovy and Sardine as Evidenced by Geostatistical and Spatial Indicators
Source: PLoS One. 2015 Aug 27;10(8):e0135808. doi: 10.1371/journal.pone.0135808 (PMC4551744; doi:10.1371/journal.pone.0135808)
Supplement: S2 Table — (DOCX) [file pone.0135808.s002.docx]

**S2 Table:** Summary table reporting the estimated spatial indicators for anchovy and sardine in each considered area (S1: Adventure Bank sector in Sicily waters, S2: Maltese Bank sector in Sicily waters, S: Sicily waters, G1: Thermaikos Gulf, G2: Thracian Sea)

|  | **Area** | **Year** | PA | SA | EA | GIC | Inertia | Isotropy | Major_patches |
| --- | --- | --- | --- | --- | --- | --- | --- | --- | --- |
| **Anchovy** | **S1** | 2002 | 0.77 | 0.8 | 0.78 | 1.23 | 1.06 | 1.06 | 2 |
|  | **S1** | 2003 | 0.69 | 0.89 | 0.94 | 0.79 | 0.82 | 0.89 | 2 |
|  | **S1** | 2005 | 0.71 | 1.1 | 1.21 | 0.55 | 0.84 | 1.25 | 2 |
|  | **S1** | 2006 | 1.24 | 1.06 | 1.01 | 0.9 | 0.59 | 0.94 | 2 |
|  | **S1** | 2007 | 0.91 | 1.04 | 1.08 | 0.89 | 0.84 | 0.91 | 3 |
|  | **S1** | 2008 | 0.95 | 0.86 | 0.81 | 1.5 | 1.4 | 0.88 | 2 |
|  | **S1** | 2009 | 1.22 | 1.11 | 1.07 | 1.51 | 1.23 | 0.86 | 2 |
|  | **S1** | 2010 | 1.49 | 1.14 | 1.11 | 0.63 | 1.23 | 1.21 | 3 |
|  | **S2** | 2002 | 1.33 | 1.38 | 1.38 | 0.83 | 1.08 | 1.09 | 2 |
|  | **S2** | 2003 | 0.98 | 0.94 | 1.01 | 1.11 | 0.76 | 0.9 | 2 |
|  | **S2** | 2005 | 0.97 | 1.53 | 1.62 | 1.09 | 1.93 | 0.98 | 2 |
|  | **S2** | 2006 | 0.92 | 0.98 | 1.01 | 1.02 | 0.78 | 0.83 | 2 |
|  | **S2** | 2007 | 0.88 | 0.85 | 0.86 | 0.93 | 0.72 | 0.89 | 3 |
|  | **S2** | 2008 | 0.77 | 0.74 | 0.67 | 0.88 | 0.66 | 1.25 | 2 |
|  | **S2** | 2009 | 0.81 | 0.75 | 0.73 | 1.05 | 1.35 | 0.74 | 2 |
|  | **S2** | 2010 | 1.32 | 0.83 | 0.71 | 1.08 | 0.72 | 1.32 | 2 |
|  | **G1** | 2004 | 0.95 | 0.89 | 0.9 | 0.96 | 0.73 | 1.23 | 2 |
|  | **G1** | 2005 | 0.93 | 0.92 | 0.93 | 1.05 | 0.82 | 0.92 | 2 |
|  | **G1** | 2006 | 1.25 | 1.16 | 1.15 | 1.09 | 1.16 | 0.78 | 3 |
|  | **G2** | 2004 | 0.71 | 0.75 | 0.76 | 0.75 | 0.45 | 1.09 | 1 |
|  | **G2** | 2005 | 0.75 | 0.59 | 0.57 | 1.13 | 1.02 | 0.93 | 2 |
|  | **G2** | 2006 | 1.29 | 1.18 | 1.18 | 0.88 | 1.11 | 1.15 | 2 |
|  | **G2** | 2008 | 1.13 | 1.17 | 1.19 | 1.12 | 1.17 | 0.84 | 2 |
| **Sardine** | **S** | 2002 | 0.69 | 0.65 | 0.64 | 0.71 | 0.53 | 1 | 2 |
|  | **S** | 2003 | 0.89 | 1.08 | 1.08 | 1.13 | 1.14 | 0.84 | 5 |
|  | **S** | 2005 | 1.05 | 1.39 | 1.42 | 0.91 | 1.33 | 1.19 | 6 |
|  | **S** | 2006 | 1.21 | 1.08 | 1.01 | 0.88 | 0.91 | 1.06 | 3 |
|  | **S** | 2007 | 0.78 | 0.81 | 0.86 | 1.15 | 0.96 | 0.95 | 3 |
|  | **S** | 2008 | 1.14 | 1.12 | 1.14 | 1.07 | 0.87 | 0.98 | 5 |
|  | **S** | 2009 | 1.06 | 1 | 1 | 1.04 | 1.41 | 0.92 | 4 |
|  | **S** | 2010 | 1.19 | 0.87 | 0.86 | 1.11 | 0.83 | 1.05 | 3 |
|  | **G1** | 2004 | 0.8 | 0.79 | 0.78 | 0.96 | 0.67 | 1.06 | 1 |
|  | **G1** | 2005 | 0.68 | 0.73 | 0.73 | 1.05 | 0.77 | 1.01 | 2 |
|  | **G1** | 2006 | 1.11 | 1.01 | 1.02 | 1.09 | 0.97 | 1.18 | 3 |
|  | **G2** | 2004 | 0.5 | 0.53 | 0.51 | 0.75 | 1.41 | 0.33 | 2 |
|  | **G2** | 2005 | 0.72 | 0.79 | 0.78 | 1.13 | 0.99 | 0.75 | 2 |
|  | **G2** | 2006 | 0.9 | 1.01 | 1.01 | 0.88 | 0.51 | 1.87 | 1 |
|  | **G2** | 2008 | 0.99 | 0.91 | 0.91 | 1.12 | 1.09 | 0.67 | 2 |
