# Supplementary material for: Interannual Changes in Biomass Affect the Spatial Aggregations of Anchovy and Sardine as Evidenced by Geostatistical and Spatial Indicators
Source: PLoS One. 2015 Aug 27;10(8):e0135808. doi: 10.1371/journal.pone.0135808 (PMC4551744; doi:10.1371/journal.pone.0135808)
Supplement: S3 Table — (DOCX) [file pone.0135808.s003.docx]

**S3 Table.** PCA scores per species. (S1: Adventure Bank sector in Sicily waters, S2 Maltese Bank sector in Sicily waters, S: Sicily waters, G1: Thermaikos Gulf, G2 Thracian Sea)

|  | **Area** | **Stand Biomass** | **PC1** | **PC2** | **PC3** | **PC4** | **PC5** |
| --- | --- | --- | --- | --- | --- | --- | --- |
| **Anchovy** | **S1** | 0.1500 | -1.3807 | -1.4917 | -0.9616 | -0.3168 | -0.6308 |
|  | **S1** | 0.7800 | -1.0175 | 0.3066 | -1.2989 | -0.5689 | -0.2207 |
|  | **S1** | 2.1700 | -0.1375 | 1.5547 | -1.7824 | -1.6492 | 0.9389 |
|  | **S1** | 1.0500 | 0.2098 | 0.5434 | -0.9113 | 0.1994 | 0.0739 |
|  | **S1** | 0.5900 | 0.6881 | -0.6642 | -1.0401 | 0.1961 | 1.5014 |
|  | **S1** | 0.3700 | 0.0390 | -2.3534 | 0.0368 | 0.5270 | -1.7473 |
|  | **S1** | 0.7200 | 1.7008 | -1.2593 | 0.3599 | 0.8187 | -1.9498 |
|  | **S1** | 2.1700 | 2.7775 | 1.1165 | -0.8726 | 1.4226 | 1.8515 |
|  | **S2** | 1.9300 | 2.3998 | 1.8295 | -0.8675 | -0.9177 | -0.4943 |
|  | **S2** | 1.0400 | -0.0304 | -0.0541 | -0.7433 | 0.4416 | -0.8970 |
|  | **S2** | 1.0900 | 3.7870 | -0.3614 | -0.0303 | -2.5371 | -1.2524 |
|  | **S2** | 0.3600 | -0.2224 | -0.8216 | -0.7395 | -0.3545 | -0.1587 |
|  | **S2** | 1.1300 | -0.3354 | -1.7348 | -1.0625 | 0.7871 | 1.9731 |
|  | **S2** | 0.5500 | -2.3974 | 0.1949 | -1.9171 | -0.1165 | 0.3276 |
|  | **S2** | 0.3500 | -0.7300 | -3.5830 | -0.1723 | -0.3295 | 0.4933 |
|  | **S2** | 1.5500 | -0.1432 | 2.5001 | -1.2154 | 2.7705 | -1.6746 |
|  | **G1** | 1.2100 | -1.4364 | 0.8919 | 1.0909 | -0.0357 | 0.7960 |
|  | **G1** | 0.4800 | -0.8637 | 0.2195 | 1.6908 | 0.2210 | 0.1108 |
|  | **G1** | 1.4800 | 1.8184 | 0.0219 | 2.1283 | 0.8697 | 0.8125 |
|  | **G2** | 0.4500 | -4.0887 | 2.1954 | 0.9357 | -1.6444 | -0.4930 |
|  | **G2** | 0.7800 | -2.6066 | -0.4345 | 2.1242 | 0.9314 | -0.2287 |
|  | **G2** | 0.8000 | 1.0728 | 2.2579 | 2.2349 | 0.0320 | 0.1806 |
|  | **G2** | 1.9700 | 0.8968 | -0.8744 | 3.0133 | -0.7468 | 0.6878 |
| **Sardine** | **S** | 0.4800 | -1.7091 | -2.1344 | -3.0968 | 0.2021 | -0.3653 |
|  | **S** | 0.8500 | 2.0817 | 0.9622 | -0.2765 | -1.0400 | 0.3762 |
|  | **S** | 1.6100 | 3.9516 | -0.1805 | -0.3140 | 1.4358 | 1.6517 |
|  | **S** | 0.7900 | 1.6208 | -0.6525 | -0.5964 | 0.2347 | -0.2082 |
|  | **S** | 0.9000 | 0.2651 | 0.6290 | -0.6418 | -0.8493 | -1.0718 |
|  | **S** | 1.3900 | 2.6008 | 0.4675 | 0.1996 | -0.3293 | -0.5845 |
|  | **S** | 0.6300 | 1.8391 | 0.9111 | -0.5711 | -0.5585 | 0.6024 |
|  | **S** | 1.3500 | 1.0408 | -0.2481 | -0.2639 | -0.4733 | -1.5860 |
|  | **G1** | 0.6700 | -2.1040 | -1.9710 | 0.1712 | -1.0631 | 0.7301 |
|  | **G1** | 0.7500 | -2.0518 | 0.2065 | 0.7067 | -1.0908 | -0.1454 |
|  | **G1** | 1.9600 | 0.3302 | 0.4018 | 1.6617 | 2.1954 | -1.1304 |
|  | **G2** | 0.3400 | -3.8115 | 2.8875 | -1.3858 | 1.4015 | 0.8915 |
|  | **G2** | 1.0300 | -1.9865 | 1.3678 | 1.0697 | 0.6535 | -0.6072 |
|  | **G2** | 1.2800 | -1.1318 | -3.6187 | 1.5695 | 0.8868 | 0.4794 |
|  | **G2** | 1.2200 | -0.9351 | 0.9717 | 1.7678 | -1.6054 | 0.9673 |
