# Supplementary material for: Interannual Changes in Biomass Affect the Spatial Aggregations of Anchovy and Sardine as Evidenced by Geostatistical and Spatial Indicators
Source: PLoS One. 2015 Aug 27;10(8):e0135808. doi: 10.1371/journal.pone.0135808 (PMC4551744; doi:10.1371/journal.pone.0135808)
Supplement: S4 Table — (DOCX) [file pone.0135808.s004.docx]

**S4 Table.** PCA scores per area.

| **Area** | **Species** | **Stand Biomass** | **PC1** | **PC2** | **PC3** | **PC4** | **PC5** |
| --- | --- | --- | --- | --- | --- | --- | --- |
| **Sicily** | anchovy | 0.1500 | -2.0064 | 0.6676 | 0.8441 | -0.0860 | -0.5918 |
|  | anchovy | 0.7800 | -1.2944 | -1.1707 | 2.0595 | -0.6109 | -1.3933 |
|  | anchovy | 2.1700 | 0.2116 | -1.8069 | 2.3312 | -2.0642 | -0.9225 |
|  | anchovy | 1.0500 | -0.1576 | -0.8659 | 1.8739 | 0.4136 | -0.0278 |
|  | anchovy | 0.5900 | -0.2767 | 0.2530 | 1.8548 | -0.6589 | -0.0457 |
|  | anchovy | 0.3700 | -1.1225 | 1.8802 | 0.9803 | 1.8169 | -0.7724 |
|  | anchovy | 0.7200 | 0.6940 | 1.3436 | 1.6216 | 2.6362 | -0.7160 |
|  | anchovy | 2.1700 | 2.2483 | -0.9649 | 2.2282 | 0.5563 | 1.3635 |
|  | anchovy | 1.9300 | 2.8077 | -1.2650 | -1.5973 | -0.1328 | -0.9694 |
|  | anchovy | 1.0400 | -0.3365 | -0.5352 | -1.8936 | 0.7252 | -0.5470 |
|  | anchovy | 1.0900 | 3.6042 | 1.7088 | -1.8592 | -0.4690 | -2.4775 |
|  | anchovy | 0.3600 | -0.8270 | 0.4156 | -0.9501 | -0.1281 | -0.5263 |
|  | anchovy | 1.1300 | -1.5334 | 0.9471 | -1.1973 | -0.7548 | 1.0181 |
|  | anchovy | 0.5500 | -2.2068 | -1.4740 | -2.1047 | -1.2603 | 0.2089 |
|  | anchovy | 0.3500 | -2.2952 | 3.1624 | -1.3068 | -0.1883 | 0.2790 |
|  | anchovy | 1.5500 | 0.4587 | -3.7102 | -1.9122 | 2.4942 | 0.5814 |
|  | sardine | 0.4800 | -2.5447 | -2.4299 | -0.3640 | -0.6639 | -0.4421 |
|  | sardine | 0.8500 | 0.4043 | 1.7294 | 0.2670 | -0.6863 | 0.7439 |
|  | sardine | 1.6100 | 3.4984 | 0.1202 | -0.2772 | -1.5582 | 1.0552 |
|  | sardine | 0.7900 | 0.6595 | -0.4290 | -0.0260 | -0.0232 | 0.7278 |
|  | sardine | 0.9000 | -1.2512 | 0.5842 | -0.6031 | 0.1486 | 0.2297 |
|  | sardine | 1.3900 | 1.0666 | 1.0480 | 0.0267 | -0.3387 | 1.8674 |
|  | sardine | 0.6300 | 0.5596 | 1.1760 | -0.0314 | -0.1044 | 0.3981 |
|  | sardine | 1.3500 | -0.3603 | -0.3846 | 0.0357 | 0.9368 | 0.9589 |
| **Greece** | anchovy | 1.2100 | 0.1703 | -1.2357 | -1.4124 | 0.2747 | -0.2900 |
|  | anchovy | 0.4800 | 0.8510 | -0.5275 | -1.4260 | -0.1258 | -0.0297 |
|  | anchovy | 1.4800 | 3.5076 | -0.4627 | -0.7042 | -0.4858 | -0.7476 |
|  | anchovy | 0.4500 | -3.8433 | -1.5595 | 0.6044 | -0.2841 | -0.8084 |
|  | anchovy | 0.7800 | -1.2358 | 1.8642 | 0.0047 | 0.2128 | 1.4658 |
|  | anchovy | 0.8000 | 1.7550 | -0.8237 | 2.4678 | -1.4987 | -0.3101 |
|  | anchovy | 1.9700 | 2.0462 | 0.5009 | 1.9878 | 1.6663 | -0.4696 |
|  | sardine | 0.6700 | -1.8957 | -1.5970 | -1.4562 | 0.2242 | -0.5692 |
|  | sardine | 0.7500 | -0.6744 | 0.1594 | -2.1214 | 0.8705 | 0.1839 |
|  | sardine | 1.9600 | 2.9200 | -0.3708 | -1.1897 | -0.9300 | 0.8376 |
|  | sardine | 0.3400 | -1.8260 | 4.0953 | -0.0336 | -1.6265 | -0.8896 |
|  | sardine | 1.0300 | -0.1914 | 1.8008 | 0.4077 | 0.2715 | 1.0914 |
|  | sardine | 1.2800 | -1.6995 | -2.8711 | 1.7513 | -0.4402 | 1.0859 |
|  | sardine | 1.2200 | 0.1161 | 1.0274 | 1.1200 | 1.8710 | -0.5504 |
